# Research Progress on the Mechanism of Acupuncture Treatment for Nonalcoholic Fatty Liver Disease

**DOI:** 10.1155/2022/5259088

**Published:** 2022-06-22

**Authors:** Bai Li, Li Fang

**Affiliations:** ^1^The Acumox and Tuina College, Shandong University of Traditional Chinese Medicine, Jinan, Shandong 250013, China; ^2^Department of Endocrinology, Shandong Provincial Hospital, Shandong University, Jinan, Shandong 250021, China

## Abstract

Nonalcoholic fatty liver disease (NAFLD) represents the most common chronic liver disease worldwide, ranging from simple steatosis and nonalcoholic steatohepatitis to fibrosis, cirrhosis, and hepatocellular carcinoma. Acupuncture is a long-established treatment in traditional Chinese medicine. In recent years, increasing evidence has pointed to the effectiveness of acupuncture in the treatment of NAFLD, and a certain degree of progress has been made in the study of related mechanisms. However, previous systematic reviews have not discussed the characteristics and the related mechanisms of acupuncture in the treatment of NAFLD. Therefore, this review synthesizes the progress in research on acupuncture in the context of NAFLD treatment by the inhibition of inflammatory responses, regulation of lipid metabolism disorder, treatment of insulin resistance, antagonization of oxidative stress injury, and interference with endoplasmic reticulum stress. Overall, we sought to highlight the latest research results, potential applications, and ongoing challenges of this therapy.

## 1. Introduction

Nonalcoholic fatty liver disease (NAFLD), now known as metabolically associated fatty liver disease, represents a common liver disease affecting approximately 30% of the global population [1]. NAFLD can develop into nonalcoholic steatohepatitis under chronic low-grade aseptic inflammatory conditions, potentially leading to fibrosis, cirrhosis, and ultimately hepatocellular carcinoma [2]. The clinical characteristics of NAFLD mainly consist of abnormal fatty deposition of hepatocytes, hepatic steatosis, severe damage with a characteristic histological appearance, hepatomegaly, and hepatic inflammatory reaction [3, 4]. NAFLD is closely linked to diseases, such as type 2 diabetes, cardiovascular disease, and chronic kidney disease; however, its specific mechanisms remain unknown. Currently, the universally recognized pathogenic mechanisms underpinning NAFLD are associated with inflammatory response, lipid metabolism, oxidative stress, endoplasmic reticulum stress, and insulin resistance (IR). However, there remains no standard therapy specifically for NAFLD, and weight loss is the only intervention that has been proven to be beneficial for patients with NAFLD [5]. Unfortunately, most people find it difficult to lose and to maintain the weight needed to cure NAFLD [6]. Therefore, it is very important to have an in-depth understanding of the pathogenesis of NAFLD and identify new alternative treatments.

Acupuncture is a common complementary and comprehensive therapy in the context of traditional Chinese medicine. It originated in China and has a history of more than 4,000 years. It can be used to promote health or to treat disease via a variety of different techniques. These include hand acupuncture, electricity acupuncture, moxibustion, and finger pressure at specific anatomical positions (acupoints) [7]. Many different studies have shown that acupuncture can effectively treat NAFLD and that it has a powerful therapeutic effect on liver fat, glucolipid metabolism, and IR [8]. In addition, acupuncture may also inhibit the progression of NAFLD by inhibiting inflammation, reducing oxidative stress, and promoting lipid metabolism in liver cells [9]. Since acupuncture is becoming more widely used, the mechanisms involved have been increasingly discussed and gradually implemented. In this review, we summarize the efficacy and potential mechanisms of acupuncture in the treatment of NAFLD and thereafter highlight its potential applications and challenges.

## 2. Acupuncture Inhibits Inflammatory Reactions in NAFLD

Inflammation plays a key role in the progression of NAFLD, and continuous inflammation may negatively impact normal physiological activities and eventually lead to liver fibrosis [10]. Low levels of chronic inflammation and lipid accumulation in the liver are considered the core pathogenic underpinnings of NAFLD [11]; the resulting lipid metabolism disorder, IR, and enteric endotoxin contribute to the production and release of proinflammatory factors tumor necrosis factor (TNF)-*α*, interleukin- (IL-) 1B, and IL-6 [12]. Therefore, inhibiting the expression of relevant inflammatory factors and maintaining the dynamic balance of inflammatory factors may represent an effective strategy for the treatment of NAFLD.

TNF-*α* plays a core role in the pathogenesis of NAFLD and is the main cytokine leading to liver injury in NAFLD [13]. Zeng Z.H. et al. [14] showed that electroacupuncture of bilateral “PI shu” (BL20), “Shen shu” (BL23), and “Ge shu” (BL17) could effectively reduce the expression level of TNF-*α* in NAFLD rats so as to further improve the inflammatory state of NAFLD and to reduce the degree of liver injury in NAFLD. In addition, early electroacupuncture of “Zu san li” (ST36), “San yin jiao” (SP6), and “Feng long” (ST40) can inhibit the expression level of TNF-*α* inflammatory factors, thus effectively regulating the inflammatory state of NAFLD [15]. IL-6 is a proinflammatory cytokine derived from the liver and adipose tissue and is associated with liver and skeletal muscle IR. IL-6 is considered the second core element in the pathophysiology of NAFLD, leading from simple fatty liver to the development of nonalcoholic steatohepatitis [16]. Chen et al. [17] stimulated acupuncture points, such as “Zu san li” (ST36), “San yin jiao” (SP6), and “Feng long” (ST40), by electroacupuncture so as to treat high-fat diet-induced NAFLD in rats and to effectively reduce the expression level of IL-6. This suggests that electroacupuncture can effectively improve the inflammatory state and liver function of NAFLD. Similarly, Yu et al. [18], respectively, intervened in high-fat diet-induced NAFLD in rats by acupuncture “Feng long” (ST40), electroacupuncture “Feng long” (ST40), or electroacupuncture “Zu san li” (ST36), demonstrating that all three methods could effectively inhibit the expression of TNF-*α* and IL-6, reduce the release of inflammatory mediators, regulate lipid metabolism disorder, and reduce the degeneration of liver lipid deposition. To reduce liver inflammation and injuries, electroacupuncture “Feng long” (ST40) is particularly potent. IL-18 is a proinflammatory cytokine that can be produced by adipocytes, macrophages, Kupffer cells, and endothelial cells, and increased IL-18 can reflect the degree of inflammation and steatosis [19]. Wang et al. [20] showed that acupuncture “Zu san li” (ST36), “Feng long” (ST40), “San yin jiao” (SP6), and “Tai chong” (LR3) in NAFLD rats induced by high-fat diet was found to reduce the expression level of IL-18 in liver tissue and serum, thus improving the degree of steatosis and inflammatory injury in liver tissue.

**Figure 1:**
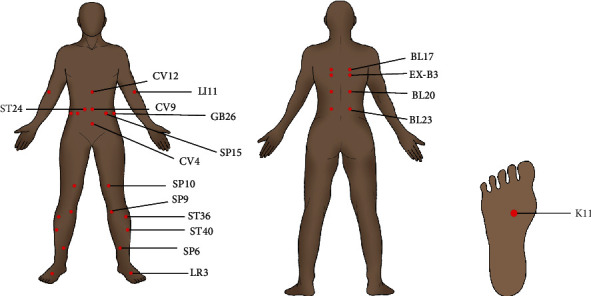
The location and distribution of the acupuncture points.

In addition, nuclear factor- (NF-) *κ*B is a key regulatory factor of liver disease, regulating a variety of pathological processes, such as inflammation, apoptosis, and cell proliferation and differentiation [21], and plays a key role in the development of chronic inflammation in NAFLD. Many studies have shown that increased NF-*κ*B activation promotes the transcription of proinflammatory cytokines, including IL-1*β*, TNF-*α*, and IL-6, resulting in inflammatory states in the body [22]. Meanwhile, Sirt1 has been proven to be involved in regulating hepatic steatosis and obesity in obese mice induced by a high-fat diet, suggesting that Sirt is closely related to NAFLD [23]. Therefore, Ma et al. [24] conducted a study based on whether electroacupuncture can participate in NAFLD treatment through the Sirt1/NF-*κ*B signaling pathway, demonstrating that electroacupuncture “Yin ling quan” (SP9), “Feng long” (ST40), and “San yin jiao” (SP6) in rats with NAFLD induced by a high-fat diet can significantly increase the expression of Sirt and decrease the levels of phosphorylated NF-*κ*B (p-NF-*κ*B), p65, pI*κ*B*α*, pI*κ*B kinase (p-IKK) *α*, and p-IKK *β*. These results suggest that electroacupuncture can reduce liver inflammation by regulating the Sirt1/NF-*κ*B pathway, thereby alleviating liver injury in NAFLD rats. In one word, this research shows that acupuncture treatment can effectively improve a NAFLD-associated chronic inflammatory state and degree of steatosis; however, so far, most studies have only discussed the inflammation-related cytokines correlated with NAFLD. There have been fewer studies focused on immune cells and the related signaling pathways. Research on acupuncture through targeted immune cells and inflammatory signaling pathways is warranted. The location and distribution of acupuncture points are detailed in Figure 1.

## 3. Acupuncture Improves Lipid Metabolism Disorder in NAFLD

The liver is a key organ for lipid metabolism. Whether it deposits or exports fat is determined as a result of fat metabolic flux through the liver. Once the homeostatic status of lipid metabolism is out of balance, excessive fat will accumulate in the liver, which may eventually lead to the development of NAFLD [25]. Lipid metabolism is affected by a variety of biological processes but mainly includes de novo adipogenesis, fatty acid intake, fatty acid oxidation, and very low-density lipoprotein (VLDL) output [26]. Once these processes turn into disorders, liver lipid metabolism will be disturbed, as characterized by the excessive accumulation of liver triglycerides (TGs). High TG deposition results thereafter in hepatic steatosis, making the liver vulnerable to proinflammatory cytokines, mitochondrial dysfunction, oxidative or endoplasmic reticulum stress, and gut microbiota attack, further leading to inflammation, apoptosis, or necrosis and fibrosis [27]. Therefore, improving lipid metabolism to reduce steatosis represents an effective strategy to block the progression of NAFLD.

Several clinical studies have demonstrated that acupuncture can reduce total cholesterol (TC), TG, and low-density lipoprotein cholesterol levels and increase high-density lipoprotein cholesterol levels in patients with dyslipidemia, thus improving the body's lipid metabolism [28] and thereby improving the progression of NAFLD resulting from lipid metabolism disorders. Meng et al. [29] assessed acupuncture at “Zu san li” (ST36), “Guan yuan” (CV4), and “Yong quan” (KI1) acupoints in the NAFLD mouse model with deficient methionine and choline, demonstrating that the levels of TG and free fatty acids (FFAs) in the liver of mice were significantly reduced, while the expression patterns of lipid metabolization-related factors were also altered. These results suggest that acupuncture can reduce lipid deposition in the liver by reducing lipid synthesis and promoting lipid metabolism. In addition, Huang et al. [30] assessed 102 patients with obesity with NAFLD treated by electroacupuncture and found that the electroacupuncture of “Feng long” (ST40) and “Zu san li” (ST36) could improve the hemorheological indices of patients, decrease TC and TG levels, and produce lipolysis, thus regulating their metabolic state. Han et al. [31] assessed the effects of acupuncture “Zu san li” (ST36), “Guan yuan” (CV4), and “Yong quan” (KI1) in the context of a NAFLD mouse model with deficient methionine and choline, demonstrating that acupuncture could reduce the accumulation of abdominal fat, inhibit the absorption of lipids in the small intestine, and downregulate the level of blood lipids in mice, thus proving that acupuncture could reduce the accumulation of abdominal fat in the case of abnormal liver metabolism and may have different beneficial effects on improving lipid metabolism. The above evidence demonstrates that acupuncture treatment can not only regulate the lipid metabolism in the liver but also affect the inhibition of lipid absorption in the small intestine to adjust the lipid metabolism of the body to improve NAFLD; however, the mechanisms underpinning “the inhibition of lipid absorption in the small intestine” remain unclear. For example, it remains unclear how this might be linked to the regulation of the intestinal microbial community in the inhibition of lipid absorption. Therefore, in a future study, acupuncture- and moxibustion-related acupuncture points may be assessed in the context of the treatment of intestinal microbial colonies and regulation of lipid metabolism. This may represent a novel treatment strategy for NAFLD.

## 4. Acupuncture Antagonizes Oxidative Stress Damage in NAFLD

A normal redox response is essential to cell survival and the maintenance of normal liver function. The excessive lipid accumulation in NAFLD affects redox reactions, and abnormal redox reactions result in an energy metabolism disorder, leading to mitochondrial dysfunction. Reactive oxygen species (ROS) levels are increased as a result of mitochondrial damage, and ROS production is not limited to the mitochondria; the endoplasmic reticulum and peroxisome also produce ROS. Excessive ROS can further accelerate lipid accumulation in hepatocytes, induce an inflammatory response in Kupffer cells, and promote fibrogenesis of hepatic stellate cells [32]. Therefore, restoring the antioxidant levels in cells and antagonizing oxidative stress damage are key to the treatment of NAFLD.

Wang et al. [33] significantly reduced the content of malondialdehyde (MDA), increased the activity levels of total superoxide dismutase (t-SOD) and glutathione peroxidase in liver tissues, and effectively improved the lipid metabolism of rats with abdominal obese NAFLD by acupuncture of bilateral “band veins.” Meng et al. [29] observed that after acupuncture of “Zu san li” (ST36), “Yong quan” (KI1), and “Guan yuan” (CV4) acupoints in NAFLD mice induced by a methionine-choline deficiency diet, the levels of oxidative stress markers 8-hydroxyldeoxyguanosine and thiobarbituric acid reactants were significantly decreased, and the expression patterns of several antioxidant enzymes, including glutathione peroxidase 1, 2, 3, glutathione synthase, and catalase, were significantly increased, thus promoting lipid metabolism. In addition, apoptosis, as the most common mode of programmed cell death, is rather frequent in the context of acute and chronic liver injury. The induction of inflammation, lipotoxicity, mitochondrial dysfunction, or other injuries can lead to the excessive apoptosis of liver cells, in turn stimulating the surrounding hepatic astrocytes and immune cells, leading to the aggravation of hepatitis and fibrosis [25]. Zhang et al. [34] assessed the acupuncture treatment of “Guan yuan” (CV4), “Zu san li” (ST36), and “Yi yu” (EX-B3) to treat high-fat diet-induced obesity model mice and found that following an acupuncture intervention, the MDA content in the liver tissues of mice decreased, the SOD activity increased, and apoptosis-related protein Bax expression levels increased; however, Bcl-2 expression levels decreased. These results suggest that acupuncture can improve liver oxidative stress and inhibit hepatocyte apoptosis, thus playing a protective role in liver cells, suggesting that the cross talk of oxidative stress and apoptosis plays an important role in obesity-induced liver dysfunction and may provide essential insights into the mechanisms of related diseases, such as NAFLD. In conclusion, the abovementioned studies show that acupuncture treatment can improve the oxidative stress state in the progression of NAFLD, promote the expression of protective antioxidants in the liver, and play a protective role in NAFLD.

## 5. Acupuncture Improves Endoplasmic Reticulum Stress in NAFLD

The ER is responsible for the correct folding of secreted proteins and transmembrane proteins. To restore homeostasis, the accumulation of misfolded or unfolded proteins leads to UR stress and the activation of the unfolded protein response (UPR) [35]. However, if endoplasmic reticulum stress is too strong or prolonged and the UPR is insufficient to relieve this stress state, abnormal endoplasmic reticulum stress interferes with liver lipid metabolism by activating fat production and limiting VLDL formation and secretion, promoting IR in the liver, and stimulating adipose tissue to indirectly act on TG accumulation. Thereafter, this results in the activation of nuclear transcription factor E2-associated factor 2 (Nrf2), C-Jun amino terminal kinase (JNK), NF-*κ*B, cyclic adenosine phosphate response element binding protein H (CREBH), and transcription factor CCAAT-enhancer binding protein homologous protein (CHOP), eventually promoting subsequent inflammation and cell death [36]. Following endoplasmic reticulum stress, active transcription factor 6 (ATF6) and the precursor form of sterol regulatory element binding protein (SREBP) in the endoplasmic reticulum membrane are translocated to the Golgi apparatus, where they are hydrolyzed and activated by site 1 protease and site 2 protease. Subsequently, the transcriptionally active ATF6 and SREBP are recruited to the nucleus. Although ATF6 plays a protective role by activating genes involved in the UPR, SREBP activates genes involved in adipogenesis and steroid production, which leads to the development of NAFLD [37].

Srebp-1c, a subtype of SREBP, is a major transcription factor upregulated by adipogenic genes in steatosis hepatocytes [38]. By increasing the expression levels of downstream factor fatty acid synthase (FAS) and acetyl-CoA carboxylase, the level of TGs in the liver is regulated; this then acts as a key regulator of fat formation [39]. Li et al. [40] significantly improved the lipid metabolism disorder of insulin-resistant rats by electroacupuncture of “Feng long” (ST40) and “San yin jiao” (SP6), speculating that the underlying mechanisms might be related to a reduction in the expression of SrebP-1c and FAS in liver tissues by electroacupuncture. Similarly, Yu et al. [41] assessed the effects of electroacupuncture of the “Feng long” (ST40) or “Zu san li” (ST36) in NAFLD in rats resulting from a high fat diet; this resulted in a benign regulatory effect on mice. The underlying mechanisms may be related to a downregulation of the expression levels of the SrebP-1C gene and protein, amelioration of endoplasmic reticulum stress, regulation of lipid metabolism disorder, and thus reduction in liver tissue inflammatory injury. Moreover, electroacupuncture of “Feng long” has a better effect. Zhang et al. [42] assessed the effects of electroacupuncture “Zu san li” (ST36) and “San yin jiao” (SP6) on the endoplasmic reticulum stress marker protein disulfide isomerase A3 (ERp57) and SrepP-1C in NAFLD rats induced by a high fat diet, finding that the lipid metabolism of rats was significantly improved by inhibiting the expression of ERp57. It can improve endoplasmic reticulum stress in the liver of NAFLD rats, thus downregulating SREBP-1C levels and reducing lipid synthesis and accumulation. In addition, later studies by this group [43] found that electroacupuncture of “Zu san li” (ST36) and “San yin jiao” (SP6), in addition to dietary control, could inhibit the expression of another endoplasmic reticulum stress marker protein, glucose-binding protein 78, thus effectively improving endoplasmic reticulum stress in NAFLD rats and contributing to the treatment of NAFLD. It has been shown that acupuncture plays an effective role in NAFLD by intervening with endoplasmic reticulum stress. However, current studies are relatively sparse with regard to the intervention of endoplasmic reticulum stress-mediated signaling pathways. Furthermore, it remains unknown whether acupuncture can affect endoplasmic reticulum stress through upregulation of the expression of Nrf2, JNK, NF-*κ*B, CREBH, and CHOP. Further research is needed.

## 6. Acupuncture Improves Insulin Resistance in NAFLD

IR, especially in adipose tissue, is considered the main driver of NAFLD [44]. The imbalance between the TG inflow rate and clearance rate leads to fat deposition in the liver, and most FFAs stored in the form of TGs are generated from the increase in lipolysis in peripheral tissues, which results from hyperinsulinemia and the increase in IR and fat production induced by dietary fat [32]. Thus, fatty deposition in the liver can lead to metabolic disorders, which further lead to excessive mitochondrial ROS production and endoplasmic reticulum stress, in addition to the eventual development of nonalcoholic steatohepatitis. As a core mechanism in many multifactorial diseases, such as NALFD, type 2 diabetes, metabolic syndrome, and obesity, IR needs to be emphasized in clinical practice.

Feng et al. [45] were the first to demonstrate that electroacupuncture could significantly reduce the levels of fasting blood glucose (FPG) and fasting insulin (FINS) and the IR index (Homeostatic Model Assessment for Insulin Resistance) while improving insulin sensitivity in rats with high-lipid-induced NAFLD by acupuncture at the “Zu san li” (ST36), “Feng long” (ST40), “San yin jiao” (SP6), and “Tai chong” (LR3) acupoints. The concentration of FFAs was significantly reduced, the vicious circle between IR and FFAs was interrupted, and the IR state was further improved. Subsequently, several researchers have demonstrated the remarkable efficacy of acupuncture in IR. For example, Dong [8] found, in a randomized controlled trial of 90 obese NAFLD patients, that acupuncture of the “Zhong wan” (CV12), “Qu chi” (LI11), “Shui fen” (CV9), “Guan yuan” (CV4), “Qi hai” (CV6), “Feng long” (ST40), “San yin Jiao” (SP6), “Tai chong” (LR3), “Xue hai” (SP10), “Hua rou Men” (ST24), and “Da heng” (SP15), combined with lifestyle control, was effective at improving liver function and glucose and lipid metabolism parameters related to human parameters, reducing the levels of FPG and FINS and HOMA-IR, and effectively improving the IR status of obese NAFLD patients. Zeng [46] stimulated acupoints, such as “Feng long” (ST40) and “Tai chong” (LR3), in NAFLD rats induced by a high fat diet by blood prick, assessing the expression patterns of FFAs, leptin (LP), adiponectin (ADP), and cAMP response element binding protein (CREP); he found that the level of ADP in the liver of rats increased significantly, while the levels of LP, FFAs, and CREP decreased significantly, thereby proving that acupuncture and purging can promote the body's ability to improve IR, effectively reduce the accumulation of liver lipids, and prevent the progression of NAFLD. In addition, two recent studies have probed the effect of electricity on IR based on the IKK/NF-*κ*B and SIRT1/ATG7 signaling pathways. Zhang [47] used an electroacupuncture treatment on the “Feng long” and “San-yin” (acupuncture points) in ZDF model rats. This was found to inhibit the activation of the IKK/NF-*κ*B pathway in the liver, kidney, and islet of rats and improve the activity of insulin substrate IRS-1, thereby improving IR status, reducing inflammatory response, and protecting liver and kidney function. IKK *β* may be a potential target of electroacupuncture for IR. Similarly, Yu [48] used an electroacupuncture treatment of the “Zu san li” (ST36), “Feng long” (ST40), “Zhong wan” (CV12), and “Guan yuan” (CV4) (acupuncture points) to treat IR rats, observing the activation of the SIRT1/ATG7 pathway, thus reducing the level of fat deposition in liver and thereafter improving insulin sensitivity and improving the IR status of rats. It is worth noting that ATG7, as an autophagy related protein, may be involved in the occurrence and development of IR-related diseases; the regulatory effect of electroacupuncture on ATG7 may be further elucidated by studies on acupuncture's involvement in the treatment of NAFLD by regulating autophagy levels. In conclusion, the above studies provide strong evidence that acupuncture can improve IR state and is a key effective way of treating NAFLD. Mechanisms of acupuncture intervention in nonalcoholic fatty liver disease are detailed in Table 1.

## 7. Discussion

In many cases, acupuncture is often defined as a “macro” treatment option rather than a “specific agent” targeting a particular disease. However, currently, such a comprehensive, multilevel, and multitarget treatment appears to be better aligned with the characteristics of NAFLD, a disease characterized by complex mechanisms. Based on current clinical evidence, acupuncture treatment has been identified as an effective approach to treat NAFLD. Through the intervention of multiple targets and multiple signaling pathways, acupuncture plays a variety of effective roles in the treatment of NAFLD, such as by inhibiting the inflammatory response, regulating lipid metabolism disorder, improving IR, antagonizing oxidative stress injury, and interfering with endoplasm reticulum stress, among others. It provides a practical scientific basis underpinning the mechanisms of acupuncture intervention in NAFLD.

However, there remain certain limitations and deficiencies to current research. First, there are many repeated and low-level controlled studies in clinical practice, which only prove the effectiveness of acupuncture treatment to a certain extent, without providing an in-depth discussion of the specific mechanisms involved. The speculations in some studies have not been followed up, thus compromising the feasibility and reliability of the conclusions as a whole. In contrast, experimental studies with a precise design remain scarce. Most studies are based on the NAFLD model induced by a high fat diet, which does not match clinical reality. The pathways and targets of research are also limited. Second, there remain no unified standards for models, acupoints, acupuncture methods (electroacupuncture or manual acupuncture), and the training levels of acupuncture practitioners, thereby affecting the experimental results to varying degrees. As NAFLD is a chronic progressive disease, the role of acupuncture treatment across various stages of NAFLD also warrants discussion. Whether different acupuncture methods and acupoints play a biased role in the intervention of different pathogenesis of NAFLD has not yet been reported, and corresponding indicators and evaluation of its timeliness and dose-sensitivity are also lacking. In addition, the complex mechanisms underpinning NAFLD have been buoyed by deepened research. Key to determining the fate of cells, autophagy and apoptosis also play an important role in NAFLD [49, 50]. Therefore, it is very important to assess whether acupuncture can alleviate NAFLD by mediating autophagy and apoptosis through related pathways. In addition, new evidence also suggests that NAFLD is closely related to intestinal flora, and acupuncture has been proved to be involved in the treatment of various diseases, including nervous system diseases, endocrine diseases, and digestive system diseases, by regulating intestinal flora [51–53]. The correlation between acupuncture and intestinal flora is also worth exploring. Although some progress has been made in acupuncture treatment of NAFLD, its mechanisms of action remain, to date, insufficiently specific and reliable. More pioneering studies and optimized acupuncture treatments are warranted to shed light on this area of clinical work and ongoing research.

## Figures and Tables

**Table 1:** Mechanisms of acupuncture intervention in nonalcoholic fatty liver disease.

Category	Research object	Acupuncture points	Mechanism of action	Reference	Year
Inhibit inflammation	Rat	Pi shu (BL20), Shen shu (BL23), and Ge shu (BL17)	Inhibits TNF-*α* expression	[14]	2014 Y
	Rat	Zu san li (ST36), San yin jiao (SP6), and Feng long (ST40)	Inhibits TNF-*α* expression	[15]	2018 Y
	Rat	Zu san li (ST36), San yin jiao (SP6), and Feng long (ST40)	Inhibits IL-6 expression	[17]	2014 Y
	Rat	Feng long (ST40), Zu san li (ST36)	Inhibits TNF-*α* and IL-6 expression	[18]	2018 Y
	Rat	Zu san li (ST36), San yin jiao (SP6), Feng long (ST40), and Tai chong (LR3)	Inhibits IL-18 expression	[20]	2013Y
	Rat	Yin ling quan (SP9), Feng long (ST40), and San yin jiao (SP6)	Regulates Sirt1/NF-*κ*B pathway	[24]	2020Y
Improve lipid metabolism disorder	Mice	Zu san li (ST36), Guan yuan (CV4), and Yong quan (KI1)	Reduces lipid synthesis and promotes lipid metabolism	[29]	2019Y
	Person	Feng long (ST40), Zu san li (ST36)	Improves hemorheological index	[30]	2021Y
	Mice	Zu san li (ST36), Guan yuan (CV4), and Yong quan (KI1)	Inhibits intestinal lipid absorption and reduces lipid accumulation	[31]	2020Y
Antagonize oxidative stress	Rat	Double side tape (GB26)	Decreases MDA content and increases t-SOD and GSH-Px activities	[33]	2019Y
	Mice	Zu san li (ST36), Guan yuan (CV4), and Yong quan (KI1)	Inhibits the expression of 8-OHdG and TBARS and increases the expression of GPX1, 2, 3, and GSS	[29]	2019Y
	Mice	Zu san li (ST36), Guan yuan (CV4), and Yi yu (EX-B3)	Decreases MDA content and increases SOD activity, as well as regulates apoptosis expression of Bax and Bcl-2	[34]	2020Y
Improve endoplasmic reticulum stress	Rat	Feng long (ST40), San yin jiao (SP6)	Inhibits the expression of SrebP-1C and downregulates FAS activity	[40]	2018Y
	Rat	Feng long (ST40), Zu san li (ST36)	Inhibits the expression of SrebP-1C	[41]	2017Y
	Rat	Zu san li (ST36), San yin jiao (SP6)	Inhibits the expression of SrebP-1C Inhibits the expression of ERp57 and SREBP-1C	[42]	2016Y
	Rat	Zu san li (ST36), San yin jiao (SP6)	Inhibits the expression of GRP78	[43]	2016Y
Improve insulin resistance	Rat	Zu san li (ST36), San yin jiao (SP6), Feng long (ST40), and Tai chong (LR3)	Decreases the levels of FPG, FINS and HOMA-IR, and FFA concentration	[45]	2008Y
	Person	Zhong wan (CV12), Qu chi (LI11), Shui fen (CV9), Guan yuan (CV4), Qi hai (CV6), Feng long (ST40), San yin jiao (SP6), Tai chong (LR3), Xue hai (SP10), Hua rou men (ST24), and Da heng (SP15)	Decreases the levels of FPG, FINS, and HOMA-IR	[8]	2020Y
	Rat	Feng long (ST40), Tai chong (LR3)	Increases ADP levels and reduces LP, FFA, and CREP levels	[46]	2018Y
	Rat	Feng long (ST40), San yin jiao (SP6)	Inhibits the IKK/NF-*κ*B pathway and increases irS-1 activity	[47]	2021Y
	Rat	Zu san li (ST36), Feng long (ST40), Zhong wan (CV12), and Guan yuan (CV4)	Activates the SIRT1/ATG7 pathway and reduces liver fat deposition	[48]	2021Y

## Data Availability

All analyses were based on previously published studies; thus, no data is required.
